# Laser therapy as treatment for oral paresthesia arising from mandibular third molar extraction

**DOI:** 10.4317/jced.56419

**Published:** 2020-06-01

**Authors:** José-de Alencar Fernandes-Neto, Thamyres-Maria-Silva Simões, Ana-Luzia-Araújo Batista, Jhonatan-Thiago Lacerda-Santos, Pettely-Thaise-de Souza-Santos Palmeira, Maria-Helena-Chaves-de Vasconcelos Catão

**Affiliations:** 1 PhD student, Graduate Program in Dentistry, State University of Paraíba - UEPB, Campina Grande / PB, Brazil; 2 Master’s student, Graduate Program in Dentistry, State University of Paraíba - UEPB, Campina Grande / PB, Brazil; 3 PhD Professor, Graduate Program in Dentistry, State University of Paraíba - UEPB, Campina Grande / PB, Brazil

## Abstract

Oral paresthesia is a localized condition of sensory abnormality that occurs in the presence of injury in one of the nerves in the region after certain dental procedures. The aim of this study was to present a case report of a patient who received low-level laser therapy as treatment for inferior alveolar nerve paresthesia due to mandibular third molar extraction surgery. A 25-year-old female patient reported lack of sensitivity for 6 months in various regions of the bucomaxillofacial complex after surgery. Laser therapy (808 ± 10nm, 100 mW, 3J per point and 30 seconds per point) was indicated twice a week. The degree of sensitivity was evaluated using a Visual Analog Scale (VAS) and with the aid of a microbrush. Prior to laser therapy, the patient reported VAS = 10, i.e., total lack of sensitivity. After 72 hours of the first session, the patient reported improvement of sensitivity in the chin (VAS = 5) and oral regions (VAS = 5), reporting recovery of sensitivity and that the area of paresthesia decreased. After 8 sessions, the patient reported total recovery of sensitivity in the chin, oral and gum regions (VAS = 0), with paresthesia being limited only to the left lower lip region and below it. After 26 sessions, the patient reported recovery of sensitivity in all affected regions (VAS = 0), with positive responses to the brush touch. Within the parameters used, laser therapy was effective in the treatment of inferior alveolar nerve paresthesia after third molar tooth extraction.

** Key words:**Lasers, paresthesia, oral surgery, low-level laser therapy.

## Introduction

Oral paresthesia occurs in the presence of injury in one of the nerves in the region, usually the inferior alveolar and / or lingual nerve in situations where they are affected by being in contact with or in close proximity to the area involved in dental procedures (1,2). Thus, it can occur in lower third molar tooth extraction (1,3), orthognathic surgery (4), local anesthesia (5), dental implant surgery (6) and endodontic treatment (7).

The main symptoms are absence or partial loss of sensitivity in the affected region, but may also present as tingling, itching, numbness or burning. These sensory abnormalities may range from mild to complete loss of sensitivity and may be devastating to the patient (1,2).

The scientific literature points out three possible actions of low-intensity lasers in the treatment of paresthesia: accelerated regeneration of the injured nerve tissue, stimulation of adjacent or contralateral nerve tissues, causing them to play the role of the injured nerve, and biomodulation of the nerve response to normality of the action potential threshold (8).

Given divergences and few studies found in current literature regarding dosimetry and irradiation protocols, the aim of this study was to report a case of a patient who received low-level laser therapy as treatment for inferior alveolar nerve paresthesia due to mandibular third molar extraction surgery.

## Case Report

**Figure 1:**
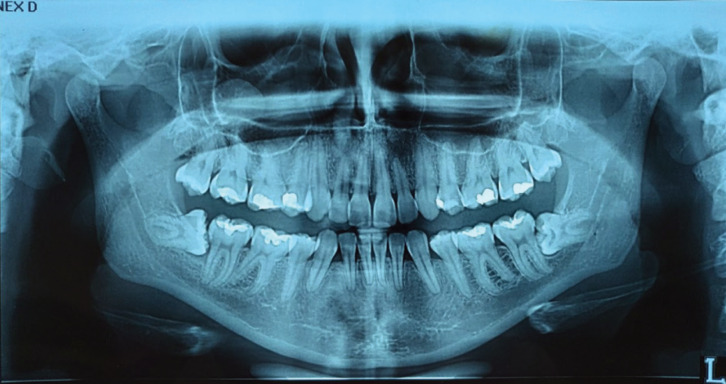
Pre-surgical panoramic radiograph, which shows impacted unerupted teeth 38 and 48 and horizontal retention. Radiographically, roots overlap the image of the mandibular canal (tooth 38).

A 25-year-old female patient attended the University Laser Therapy Clinic reporting lack of sensitivity in various regions of the bucomaxilofacial complex. At first consultation, during anamnesis and physical examination, the patient reported having had surgery for lower third molar extraction 6 months ago (Fig. 1), and since this procedure, she found complete lack of sensitivity in the lower lip (left side) and below it, lower gum (region from tooth 33 to 31) and in the chin and left oral regions.

After 3 months of surgery, the patient reported oral treatment for 30 days, 3 times a day, with 2.5 mg cytidine disodium phosphate + 1.5 mg uridine trisodium triphosphate + 1.0 mg hydroxocobalamin acetate capsules, obtaining no satisfactory results, thus persisting total lack of sensitivity in these regions. After evaluation, it was concluded that it was a case of inferior alveolar nerve paresthesia after left lower third molar extraction, and thus treatment with low intensity laser therapy twice a week with 72 hours interval between sessions was indicated.

**Figure 2:**
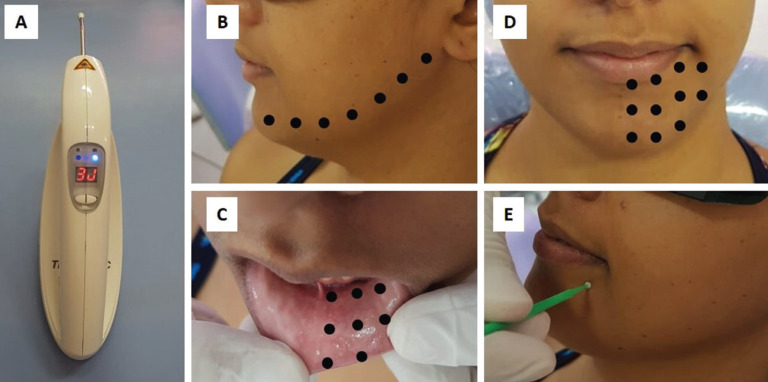
A) Laser apparatus used. B, C and D) Extraoral and irradiation pathways and points, respecting the distance of approximately 1 cm between points. E) Microbrush touch sensitivity test.

**Figure 3:**
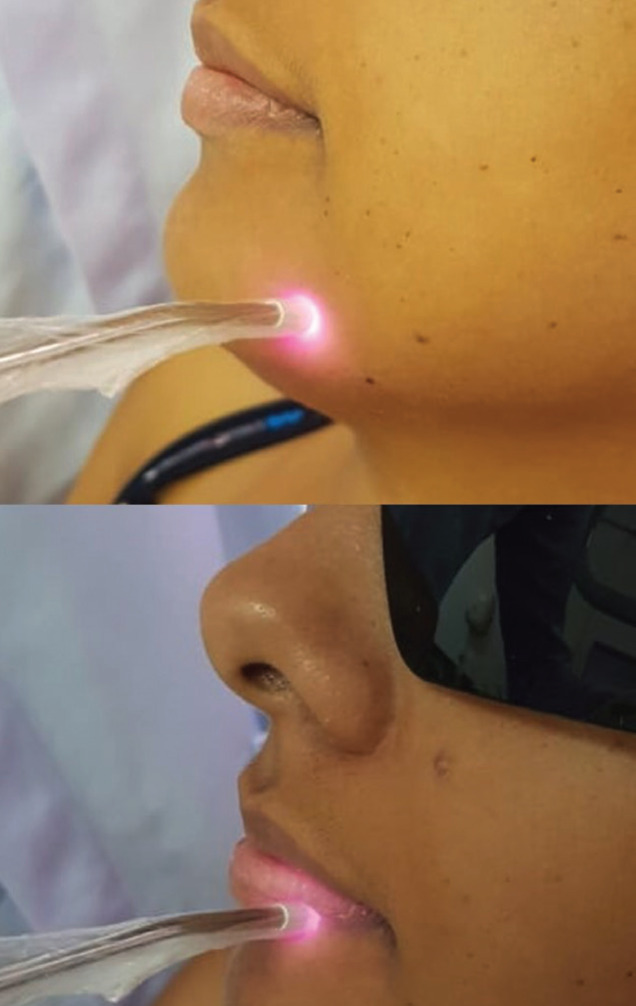
Infrared laser irradiation in the paresthesia region.

The laser device used was Therapy EC® DMC (São Carlos, SP, Brazil), with the following dosimetry: 808 ± 10nm (infrared laser), 100 mW, 3J per point and 30 seconds per point (Fig. 2). Irradiation was always performed punctually, in direct contact with the region, in continuous mode and with the laser beam perpendicular to the tissue (Fig. 3). Prior to irradiation, the patient’s skin and oral mucosa were dried with sterile gauze to remove saliva, sweat and / or cosmetic products in order to avoid interference with the laser beam.

Irradiation points in each session were: 1 point in the retromolar trigone region, vestibular sulcus (inferior alveolar nerve and mentonian nerve pathway), labial mucosa, inferior alveolar nerve pathway through the lingual ridge, extraoral pathway of the inferior alveolar nerve and chin region, respecting the distance of approximately 1 cm between points (Fig. 2). All biosafety standards have been properly followed.

The degree of sensitivity was evaluated using a 10 cm Visual Analog Scale (VAS), with 0 (zero) indicating no symptoms (normal sensitivity) and 10 (ten) indicating total lack of sensitivity in the region. In addition, a single operator, with the help of a microbrush, touched the affected and contralateral regions of the face and asked about the presence or absence of sensitivity (Fig. 2). On the first day, before the first laser therapy session, the patient reported VAS = 10, i.e., total lack of sensitivity in all regions affected by paresthesia. Microbrush tests were performed, which were negative to the touch in disorder regions and positive in the normal and opposite regions of the face.

After 72 hours and before the second laser therapy session, the patient reported substantial improvement in chin (VAS = 5) and oral (VAS = 5) sensitivity, reporting that sensitivity was returning, and that the area of paresthesia decreased. After 8 sessions, the patient reported total recovery of chin, oral and gum sensitivity (VAS = 0), with paresthesia being limited only to the left lower lip region and below it (VAS = 10).

After 12 sessions, the patient also reported lack of sensitivity in the left lower lip region and below it (VAS = 10) and that the other previously affected regions remained normal. After 16 sessions, the patient began to feel the region below the lower lip (VAS = 0), with paresthesia restricted only to the left side of the lower lip.

After 20 laser therapy sessions, the patient reported that paresthesia was restricted to the lower lip vermilion border on the left side at a single point in the region (VAS = 10). All other previously affected regions were normal to the brush touch. After 26 sessions, the patient reported recovery of sensitivity in all affected regions (VAS = 0), with positive and normal responses to the brush touch. Based on these results, the patient was reevaluated 7, 24 and 30 days after the end of treatment, with the same tests, and no areas with lack of sensitivity were observed and the patient reported complete satisfaction with the therapy performed. The patient signed an informed consent form authorizing the use of data for publications for scientific purposes.

## Discussion

Given divergences and few studies found in current literature regarding dosimetry and irradiation protocols, it is worth pointing out that the parameters used for this case were efficient.

Santos *et al* (9) evaluated the effect of laser therapy (780nm, 157.5 J/cm2, 90 seconds / point and 5 sessions at intervals from 3 to 4 weeks) on the sensorineural recovery of patients undergoing oral surgeries and observed significant improvement across sessions in patients with short postoperative period of 30 days and patients with persistent sensory abnormalities in the late postoperative period from 6 months to 1 year; however, the first group of patients showed the best results. Therefore, the idea that time is of extreme importance in the patient’s response is plausible, and that the dentist should indicate laser therapy as soon as he / she notices or suspects sensorineural damage.

In a retrospective study, Oliveira *et al.* (10) evaluated the effects of laser therapy (808nm or 660nm, 100mW, 100 J/cm2, 2.8J and 28 seconds/point) on the recovery of nerve sensitivity after minor and orthognathic oral surgery and observed that recovery was correlated with patient`s age and interval between surgery and start of laser therapy, concluding that infrared laser therapy can positively affect sensitivity after oral surgery.

Therefore, if the patient starts treatment soon after surgery or emergence of paresthesia, the number of laser therapy sessions might be lower. However, laser therapy was effective even in this case, where sensory abnormality was persistent in a late postoperative period of 6 months.

The patient reported no positive results from the drug combination used to restore peripheral nerve injuries used before laser therapy treatment and traditionally prescribed in cases of paresthesia. Therefore, laser therapy is a treatment modality that should be considered and understood by professionals.

It is also important to highlight that the major advantages of laser therapy in these types of cases are: absence of contraindication and side effects, totally noninvasive and painless treatment and most treated patients show significant improvements (4,11,12). In addition, laser therapy can be used alone or in combination with traditional treatments but should always be safely performed by trained professionals.

## Conclusions

Within the parameters used, laser therapy was effective in the treatment of inferior alveolar nerve paresthesia due to third molar tooth extraction. In addition, laser therapy was well accepted by patients and proved to be a painless, noninvasive alternative with no side effects.
